# HMGA2 Supports Cancer Hallmarks in Triple-Negative Breast Cancer

**DOI:** 10.3390/cancers13205197

**Published:** 2021-10-16

**Authors:** Behzad Mansoori, Mikkel Green Terp, Ali Mohammadi, Christina Bøg Pedersen, Henrik Jørn Ditzel, Behzad Baradaran, Morten Frier Gjerstorff

**Affiliations:** 1 Immunology Research Center, Tabriz University of Medical Sciences, Golghasht St., Tabriz 51666-14731, Iran; b.mansoori_lab@yahoo.com (B.M.); baradaranb@tbzmed.ac.ir (B.B.); 2 Department of Cancer and Inflammation Research, Institute for Molecular Medicine, University of Southern Denmark, J. B. Winsløws Vej 25, 3, DK-5000 Odense C, Denmark; mterp@health.sdu.dk (M.G.T.); amohammadi@health.sdu.dk (A.M.); cbpedersen@health.sdu.dk (C.B.P.); hditzel@health.sdu.dk (H.J.D.); 3 Aging Research Institute, Physical Medicine and Rehabilitation Research Center, Tabriz University of Medical Sciences, Golghasht St., Tabriz 51656-65811, Iran; 4 Department of Oncology, Odense University Hospital, DK-5000 Odense C, Denmark; 5 Academy of Geriatric Cancer Research (AgeCare), Odense University Hospital, DK-5000 Odense C, Denmark

**Keywords:** HMGA2, triple-negative breast cancer, NF-kB, IL-6, IL-8, STAT3

## Abstract

**Simple Summary:**

Triple-negative breast cancer grows and spreads faster compared to other types of invasive breast cancer and has limited treatment options and subsequently worse prognosis. High mobility group A 2 (HMGA2) protein is widely expressed in undifferentiated cells including cancer cells. HMGA2 is a DNA binding protein involved in DNA architecture and the functional regulation of DNA. It was reported that HMGA2 is aberrantly regulated malignant signaling in different types of cancers. In this study, we aim to reveal the role of HMGA2 in TNBC biology and demonstrate a link between HMGA2-mediated cancer phenotypes and the regulation of NF-kB/IL-6/STAT3 signaling.

**Abstract:**

Triple-negative breast cancer (TNBC) is an aggressive subtype of breast cancer that exhibits a high proliferation rate and early metastasis leading to a poor prognosis. HMGA2 is a DNA binding transcriptional regulator implicated in tumorigenesis. Here, we demonstrate that the *HMGA2* promoter is demethylated in TNBC tumors, leading to increased expression of HMGA2 at both mRNA and protein levels. Importantly, high HMGA2 levels in TNBC tumors are correlated with poor prognosis. To detail the role of HMGA2 in TNBC development and progression, we studied its effect on core cancer phenotypes. Stable knockdown of HMGA2 in TNBC cells revealed that HMGA2 may support cell proliferation, cell migration and invasion. In addition, HMGA2 knockdown decreased cancer stem cell (CSC) features. Importantly, we found that silencing HMGA2 inhibited NF-kB signaling and lead to decreased expression of the downstream molecules IL-6 and IL-8 and reduced STAT3 pathway activation. Our results demonstrate that HMGA2 supports cancer hallmarks in TNBC and may represent a promising target for TNBC treatment.

## 1. Introduction

According to WHO, breast cancer is the most common cancer and the second leading cause of death in women. Based on molecular profiling, breast cancer is divided into five subtypes, i.e., normal breast-like, luminal A, luminal B, HER2-enriched, and triple-negative, each of which have a very different prognosis and treatment options [1]. Of those diagnosed with breast cancer, 15–20% are classified as triple-negative breast cancer (TNBC) [2], which is identified by the absence of an estrogen receptor (ER) and a progesterone receptor (PR) and does not make too much of epidermal growth factor receptor type 2 (Erbb2/HER2) [3]. TNBC is generally very aggressive, with a high proliferation rate and early metastasis, leading to a poor prognosis [4].

The hallmarks in cancer constitute an organizing principle whereby the complexities of cancer can be dissected into distinct biological abilities, including sustained proliferation, induced angiogenesis, evaded growth suppression, invasion and metastasis to the tissues, self-renewal and immortality, resistance to cell death mechanisms, altered cellular energetics and evaded from the immune response. Underlying these hallmarks are genome instability and epigenetic deregulation, which changes the transcriptional and proteomic landscapes to expedite their acquisition [5]. HMGA2 (High Mobility Group AT-Hook 2) is an intranuclear non-histone DNA binding protein that functions as a DNA architectural and transcriptional regulator and is involved in several biological processes, including cell division, DNA damage/repair, chromatin organization and apoptosis [6,7]. HMGA2 is abundantly expressed in early embryonic growth and its expression level decreases in stages until birth [8]. It was reported that *HMGA2* expression is upregulated in most malignancies, including lung, colorectal [9] and breast cancer [6,10]. Overexpression of HMGA2 in cancer alters the cellular phenotype from epithelial to mesenchymal [11,12] and also modifies cellular differentiation processes. The latter involves HMGA2-mediated support of cancer cell colonization, spheroid formation and cancer stem cell markers such as ALDH, SOX2, OCT4, CD133, CD44 [11,13]. In our recent studies of the role of HMGA2 in breast cancer, we demonstrated its overexpression in this cancer type and further showed that HMGA2 supports important oncogenic features in these cancer cells [6,11,14].

Here, we focus on the role of HMGA2 in the biology of TNBC and demonstrate a link between HMGA2-mediated cancer phenotypes and regulation of NF-kB/IL-6/STAT3 signaling.

## 2. Materials and Methods

### 2.1. Bioinformatic Analysis of Human Patient Samples

HMGA2 promoter methylation (*n* = 591), mRNA (*n* = 833) and protein (*n* = 108) clinical tumor samples from different breast cancer subtypes including Liminal, HER2+, and TNBC were obtained from The Cancer Institute’s Clinical Proteomic Tumor Consortium (CPTAC) and The Cancer Genome Atlas (TCGA) repository, respectively and compared with normal breast tissue [15]. The Kaplan Meier curve of patient survival probability in Liminal (*n* = 841), HER2+ (*n* = 156) and TNBC (*n* = 161) subtypes were generated using the Kaplan–Meier Plotter database as previously published [16]. HMGA2 mRNA expression level in different TNBC cell lines was analyzed from the online dataset GSE-12777 (GPL-GPL570). The role of methylation in HMGA2 expression was evaluated by analyzing the online dataset GSE-150298 (GPL-13497). The oncoprint results performed by cBioporatal database using invasive breast cancer (TCGA, Nature2012) dataset (*n* = 463). The tissues classified according to the PAM50 subtypes. To identify differentially expressed genes (DEGs) between *HMGA2* knockdown and negative control groups, the online dataset GSE118857 (GPL6884 platform) was used for the analysis. DEGs were selected by the criteria of two-fold difference and a significance of *p* ≤ 0.01 from 28,742 genes. The volcano plot was prepared by extracting false discovery rate (FDR) and Log fold change (Log FC). The top 50 genes were ranked according to their *p*-value and enriched by the method described by Chen et al. [17] using Gene Set Enrichment Analysis software (GSEA, Broad Institute) and MSigDB [18].

### 2.2. Cell Culture and Lentiviral Transductions

TNBC breast cancer cell lines, including MDA-MB-231 and Cal-51, were obtained from the ATCC, Middlesex, UK. All cell lines were cultured in DMEM, complemented with 10% FBS (Invitrogen, Carlsbad, CA, USA), and 1% penicillin–streptomycin. Before the experiment, the cell lines were tested for the presence of mycoplasma with MycoAlert (Lonza, Verviers, Belgium).

For lentiviral transduction, HEK293T cells were transfected with 3rd generation packaging plasmids including pVSVG, pRSV-Rev and pMDL g/p RRE (kindly provided by Didier Trone through Addgene, Cambridge, MA, USA) and shERWOOD UltramiR Lentiviral Inducible shRNA target gene set for gene HMGA2 or non-targeting shRNA (TransOMIC Technologies, Huntsville, AL, USA), using Lentifectin according to the manufacturer’s recommendations. shRNAs identifiers were: HMGA2-shRNA1 (ULTRA-3398612), HMGA2-shRNA2(ULTRA-3398613), and HMGA2-shRNA3 (ULTRA-3457905). After 72 h, media containing lentivirus was collected, filtered and stored at -80 degrees.

TNBC cells were seeded at a density of 20,000 cells/cm^2^. The next day, the cells were transduced by adding media containing lentivirus particles and 5 μg/mL of polybrene for 24 h, after which the lentiviral-positive cells were selected by addition of 0.5 μg/mL of puromycin.

### 2.3. qRT-PCR Gene Expression

Total RNA was extracted using Trizol reagent (Ambion, Austin, TX, USA). Next, cDNA was synthesized with 5 µg of total RNA using the Maxima™ H Minus First Strand cDNA Synthesis Kit (Thermo Fisher Scientific Inc.). Then qRT-PCR was performed on an Applied Biosystems Step-One-plus system (Applied Biosystems, Foster City, CA, USA) using high ROX SYBR Premix (Ampliqon, Denmark). All experiments were performed in triplicates. Pumilio Homolog 1 (*PUM1*) was used as a reference gene to normalize target gene expression. Specific primers for detecting *HMGA2* obtained from Sigma Aldrich were: forward sequence 5′-AGCTCAAAAGAAAGCAGAAG-3′ and reverse sequence 5′-CCCTTCAAAAGATCCAACTG-3′, and *IL-6*, *IL-8* primers were purchased from Qiagen [QuantiTech Primer for qRT-PCR Assays, QT00083720 (IL6), QT00000322 (IL8)]. Relative quantification in mRNA changes was determined by the 2^−ΔΔCt^ method.

### 2.4. Western Blot

Total protein was extracted using RIPA lysis buffer (50mM Tris-HCl, 150nM NaCl, 1% IGE-PAL, 0.5% sodium deoxycholate, 0.1% SDS, protease, and phosphatase inhibitor cocktail (Roche Applied Science, Bavaria, Germany)). A total of 25 µg of protein was loaded into each well of SDS-PAGE gels with 4% stacking and 10% running buffer (Bio-Rad Laboratories, Inc.). The proteins were blotted onto the PVDF membrane (Bio-Rad Laboratories, Inc.) and blocked with blocking buffer (TBS buffer contained 0.5% Tween-20 and 4% skim milk). The membrane was then incubated with primary mouse monoclonal antibody against HMGA2 (Abcam, ab97276), NF-kB-P65 (Cell signaling technology, 8242T), p-NF-kB-P65 (Cell signaling technology, 3031S), STAT3 (Cell signaling technology, 9139S), p-STAT3 (Cell signaling technology, 9145S), and GAPDH (Santa Cruz Biotechnology, SC-32233) overnight at 4 °C on a shaker. Next, the membrane was incubated with secondary goat anti-mouse or mouse anti-rabbit antibodies conjugated with HRP (Dako Corporation) on a shaker for 1h at room temperature. The protein bands were developed with ECL reagent (Bio-Rad Laboratories, Inc.) and visualized on autoradiography films.

### 2.5. Immunohistochemical Staining

TNBC and normal tissue microarrays BR1301 and BN0811 (Biocat) were deparaffinized and treated with 1.5% H_2_O_2_ in Tris-buffered saline (pH 7.5) for 10 min to block peroxidase activity. Samples were then washed in TNT buffer (0.1 m Tris, 0.15 m NaCl, 0.05% Tween-20, pH 7.5) and heated to 100 °C in T-EG buffer (10 mm Tris, 0.5 mm EGTA, pH 9.0) for 48 min. This was followed by incubation with rabbit anti-HMGA2 (D1A7; Cell Signaling) diluted in antibody diluent (S2022, DAKO Cytomation, Glostrup, Denmark) for 1 h at room temperature. Then, samples were washed with TNT, incubated with OptiView DAB (Roche), washed again and incubated with 3,3′-diaminobenzidine (DAB)+ substrate-chromogen for 10 minutes. After another wash with H_2_O, tissues were counterstained with Mayers hematoxylin before mounting in AquaTex (Merck Inc., Whitehouse Station, NJ, USA).

### 2.6. ELISA Assay

To measured IL-6 and IL-8 production after HMGA2-knockdown, were measured in the supernatants using IL-6 and IL-8 human uncoated ELISA kit (Invitrogen).

### 2.7. Crystal Violet Assay

Cells (2 × 10^4^) were seeded into 24-well plates and incubated at 37 °C in 5% CO_2_ for 48 h. The cells were then cells fixed and stained with 0.5% crystal violet, 25% methanol solution at RT for 20 min. After washing, the cells were solubilized in sodium citrate buffer and crystal violet concentration measured absorbance at OD 570 nm.

### 2.8. EDU Assay

Cell proliferation was measured by Click-iT™ EdU Cell Proliferation Assay Kit. Briefly, 2 × 10^5^ cells were seeded into 6-well plates and treated with doxycycline (1 µg\mL) for 48 h. The cells were then grown for 3h in a medium containing EdU and subsequently fixed in 10% neutral buffered formalin. Incorporated EdU was labeled with the Alexa Fluor^®^ 488 azide probe according to the manufacturer’s instructions.

### 2.9. Cell Cycle FACS Assay

The percentage of cells in different cell cycle phases was evaluated using PI DNA staining in combination with flow cytometry. Briefly, the cells were harvested by trypsin-EDTA and washed twice with phosphate buffer. Cells were then fixed with ice-cold 75% ethanol by adding it drop by drop to the cell pellet while vortexing and incubated at −20 °C overnight. The next day, the cells were washed twice with PBS and stained with propidium iodide solution (50 ug/mL propidium iodide, 1% RNase A in PBS) at RT for 10 min with light protection. Finally, the cells were analyzed using the LSR II flow cytometry instrument (Becton-Dickinson, Franklin Lakes, NJ, USA). Different phases of the cell cycle were analyzed by FlowJO software (FlowJo LLC, Ashland, OR, USA).

### 2.10. Migration Assay

TNBC cell migration was evaluated using scratch wound healing assays. A total of 2 × 10^5^ cells were seeded in 6-well cell culture plates. The scratch was applied to monolayer cells and the wound area was measured by ImageJ software (http://rsb.info.nih.gov/ij/, accessed on 10 October 2020) after 0 and 48 h. The level of migration was calculated as width of wound area at 48 h relative to that at the 0 h timepoint.

### 2.11. Invasion Assay

For invasion assays, 5 × 10^3^ cells were re-suspended in RPMI-1640 without FBS and seeded in the transwell chambers precoated with Matrigel (Corning Inc., Corning, NY, USA). After 48 h, the membrane was stained with crystal violet dye. The top surface of membranes was cleaned, and the cells that invaded through the 8-µm pores were counted using ImageJ software. To account for proliferation effects, the numbers of migrated cells were normalized to the number of cells on the top of the membranes.

### 2.12. Colony Formation Assay

Cells (5 × 10^3^) were seeded per well of 6-well cell culture plates and maintained in media contained 10% FBS for 2 weeks. Then, the colonies were fixed and stained with crystal violet solution (0.5% *w*/*v*). The colonies number was determined using ImageJ.

### 2.13. Spheroid Assay

Cells (1 × 10^3^) were cultured per well of 96-well cell culture plates in a serum-free medium (Gibco, UK) contained Matrigel (200 mg/mL). The size and number of mammospheres were measured after 8 days using ImageJ.

### 2.14. CD44 and CD24 FACS Assay

MDA-MB-231 and Cal-51 cells were harvested using 5 mM EDTA and washed with prechilled FACS buffer (10% BSA in PBS). Then, 2 × 10^5^ cells were then re-suspended in 100 μL DMEM containing 10% FBS. APC-conjugated mouse anti-human CD44 monoclonal antibody (9 μL, Miltenyi Biotec Inc., Köln, Germany) and FITC-conjugated mouse anti-human CD24 monoclonal antibody (8 μL, Miltenyi Biotec Inc., Germany) were added to the cells and then incubated at 4 °C for 1 h in the dark with mild agitation. Next, the cells were rinsed thrice with chilled FACS buffer. CD44 and CD24 levels were determined using the BD LSR II flow cytometry system (BD Biosciences). Cells stained with FITC- and PE-conjugated isotype control antibodies (Miltenyi Biotec Inc., Germany) were used as positive control and unstained cells as a negative control.

### 2.15. Xenograft Model

Cal-51 cells (2 × 10^6^) stably transduced with doxycycline (dox)-inducible scramble or HMGA2 shRNA encoding plasmids were injected subcutaneously in 8-week-old female NOG CIEA (NOD.Cg-*Prkdc^scid^ Il2rg^tm1Sug^*/JicTac) mice (Taconic). Drinking water supplemented with 50 mg/mL doxycycline and 5% sucrose was supplied to mice to induce shRNA expression in tumor cells. Tumor volumes were measured at the indicated time points using a vernier caliper. The mice were sacrificed five weeks after tumor initiation and the weight and size of excised tumors were evaluated. Lungs and livers were fixed in formalin and paraffin-embedded and stained for pan-cytokeratin (anti-CKKL1; 1:30) to identify metastasis. Animal experiments were approved by the Experimental Animal Committee of The Danish Ministry of Justice and were performed at the animal core facility at the University of Southern Denmark (ethical code number 2021-15-0201-00843). Animals were euthanized if they showed any adverse signs of disease, including weight loss, paralysis, thymus dysfunction or general discomfort. Mice were housed under pathogen-free conditions with ad libitum food and water.

### 2.16. Statistical Analysis

All experimental data were analyzed by Graphpad Prism software (San Diego, CA, USA) and bioinformatic analysis was performed using R software (version 3.5.1, R Foundation for Statistical Computing). All values are represented as means of at least three independent experiments ± standard deviation (SD). The parametric values including in vitro gene and protein expression, proliferation, invasion, migration, ELISA, spheroid assays and colony formation were analyzed by a two-tailed Student’s *t*-test and nonparametric values include all the clinical and animal samples analyzed by Mann–Whitney U test. *p*-value < 0.05 was considered statistically significant.

## 3. Results

### 3.1. HMGA2 Is Overexpressed in Triple-Negative Breast Cancer

**Figure 1:**
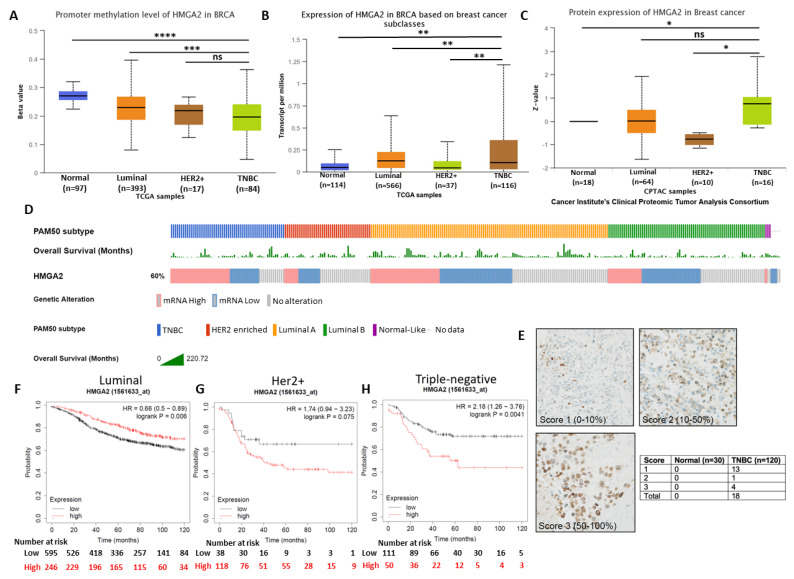
HMGA2 overexpression reduced the survival rate in TNBC patients. (**A**) *HMGA2* promoter methylation was decreased in TNBC subclasses compared to luminal breast cancer and normal tissues. HMGA2 (**B**) mRNA and (**C**) protein expression was increased in TNBC compared to other breast cancer subclasses from TCGA and the Cancer Institute’s Clinical Proteomic Tumor Consortium (CPTAC) samples, respectively. HMGA2 overexpression was shown in more than 50% TNBC patients and overall survival of each patient presented as separate row above the HMGA2 expression (**D**). Representative immunohistochemistry staining for HMGA2 in normal (*n* = 30) and TNBC tissues (*n* = 120) (**E**). Kaplan Meier survival analysis compares breast cancer patients with luminal (**F**), HER2+ (**G**) and TNBC (**H**) subtypes exhibiting HMGA2 low (black line) versus HMGA2 high (red line) expression. The analysis showed that low HMGA2 in patients with luminal breast cancer was associated with poor overall survival, as was low HMGA2 in patients with HER2+ breast cancer and TNBC. * *p* < 0.05, ** *p* < 0.01, *** *p* < 0.001, and **** *p* < 0.0001.

To investigate the gene promoter methylation status and expression level of *HMGA2* in different breast cancer subtypes, we analyzed publicly available TCGA breast cancer datasets. This demonstrated that *HMGA2* promoter methylation was significantly lower in the TNBC and HER2+ breast cancer tissues compared to luminal breast cancer tissues (*p* = 9.43 × 10^−4^) and normal tissues (*p* = 4.78 × 10^−11^) (Figure 1A). Accordingly, analysis of the RNA expression showed HMGA2 significantly increased in TNBC subclasses compared to Normal (*p*-Value = 2.76 × 10^−3^), luminal (*p*-Value = 4.94 × 10^−3^) and HER2+ (*p*-Value = 3.46 × 10^−3^) subclasses (Figure 1B). Besides, HMGA2 protein expression in breast cancer samples from the Cancer Institute’s Clinical Proteomic Tumor Consortium (CPTAC) showed that the HMGA2 protein levels were increased in TNBC samples compared to normal tissues (*p* < 0,05) (Figure 1C). HER2-positive breast cancers exhibited reduced HMGA2 levels, while no change was observed for the luminal subgroup. To confirm the HMGA2 mRNA expression in different breast cancer subtypes, we used the tab OncoPrint to analyze the data based on an invasive breast cancer (TCGA, Nature 2012) dataset. The result showed HMGA2 overexpressed in more than 50% samples in TNBC subtypes, however, in Luminal A and B subtypes are less (Figure 1D). The expression of HMGA2 proteins was assessed by immunohistochemistry on normal breast epithelium (i.e., normal adjacent tissue/hyperplasia; 30 tissues) and TNBC tissues (120 tissues) (Figure 1E and Supplementary Table S1). As expected, no staining was found in normal tissues. The HMGA2 staining in tumors was scored according to the number of positive cells. Out of 18 tissues, 13 were in score 1 (0–10% positive cells), 1 was in score 2 (10–50% positive cells) and 4 were in score 3 (50–100%) (Figure 1E). This analysis confirmed that HMGA2 is induced in expression in TNBC compared to normal breast epithelial. It also demonstrated that HMGA2 expression is highly heterogeneous in TNBC tumors. Importantly, Kaplan Meier survival plots revealed a correlation between high *HMGA2* expression and increased overall survival in the luminal subclass (HR = 0.65, log rank *p* = 0.006) (Figure 1F), whereas high *HMGA2* expression tended to be associated with decreased survival in HER2+ patients (HR = 1.74, log rank *p* = 0.075) (Figure 1G) and was significantly associated with decreased survival of TNBC patients (HR = 2.18, log rank *p* = 0.004) (Figure 1H). These results suggested a specific role for HMGA2 in oncogenesis of TNBC patients.

To further investigate the role of HMGA2 in TNBC, we generated TNBC cell line models with stable knockdown of *HMGA2* to enable investigation of the role of HMGA2 over extended periods in tumor formation and metastasis using mice xenograft models.

### 3.2. HMGA2 Supports Hallmark Phenotypes of TNBC Cells

**Figure 2:**
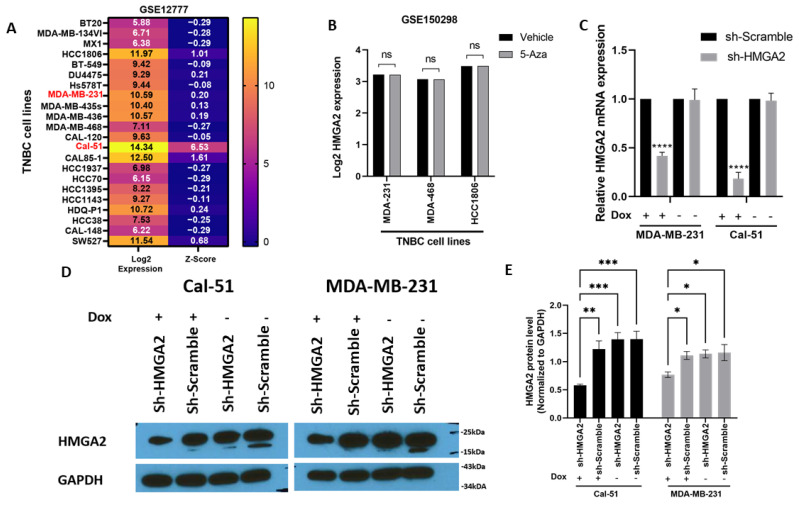
Stable knockdown of *HMGA2* expression in TNBC cell lines. HMGA2 mRNA expression level from GSE12777 dataset showing HMGA2 expression in TNBC cell lines (**A**). HMGA2 expression after 5-Azacytidine treatment from dataset GSE150298 did not show changes after treatment in any of MDA-MB-468, MDA-MB-231 and HCC1860 cell lines (**B**). MDA-MB-231 and Cal-51 TNBC cell lines were stably transduced with plasmids encoding a doxycycline-inducible HMGA2 specific shRNA (shHMGA2) or a scrambled shRNA (shScrambled). Treatment of the cells with 1 µg/mL doxycycline (Dox) to induce expression of HMGA2 shRNAs decreased HMGA2 mRNA (**C**) and protein (**D**,**E**) levels. The results are shown as mean ±SD (*n* = 3) of shHMGA2 versus the shScramble group, * *p* < 0.05, ** *p* < 0.01, *** *p* < 0.001, **** *p* < 0.0001 by *t*-test.

To select TNBC cell lines for investigating the cellular functions of HMGA2, we used an online dataset (Figure 2A). This showed that HMGA2 mRNA expression was high in Cal-51 cells and moderate in MDA-MB-231 cells and therefore these cell lines were used for our further experiments. To further understand the role of methylation in the HMGA2 level, we turned to an online dataset that treated the TNBC cell lines with DNA methyltransferase inhibitor (5-Azacytidine). The results showed TNBC treatment did not affect HMGA2 expression in MDA-MB-468, MDA-MB-231 and HCC1860 cell lines (Figure 2B). To analyze the role of HMGA2 in TNBC tumorigenesis, we established two cell line models with inducible knockdown of *HMGA2* based on the TNBC cells lines MDA-MB-231 and Cal-51. A pool of doxycycline-inducible HMGA2-specific shRNAs or non-targeting shRNA was stably inserted into MDA-MB-231 and Cal-51 cells using lentiviral transduction. Upon doxycycline treatment, *HMGA2* expression was reduced by 58.12 ± 3.5% and 81.66 ± 6.5% in MDA-MB-231 and Cal-51 cells, respectively (Figure 2C). shRNA-mediated reduction of HMGA2 was confirmed by Western blotting (Figure 2D,E).

**Figure 3:**
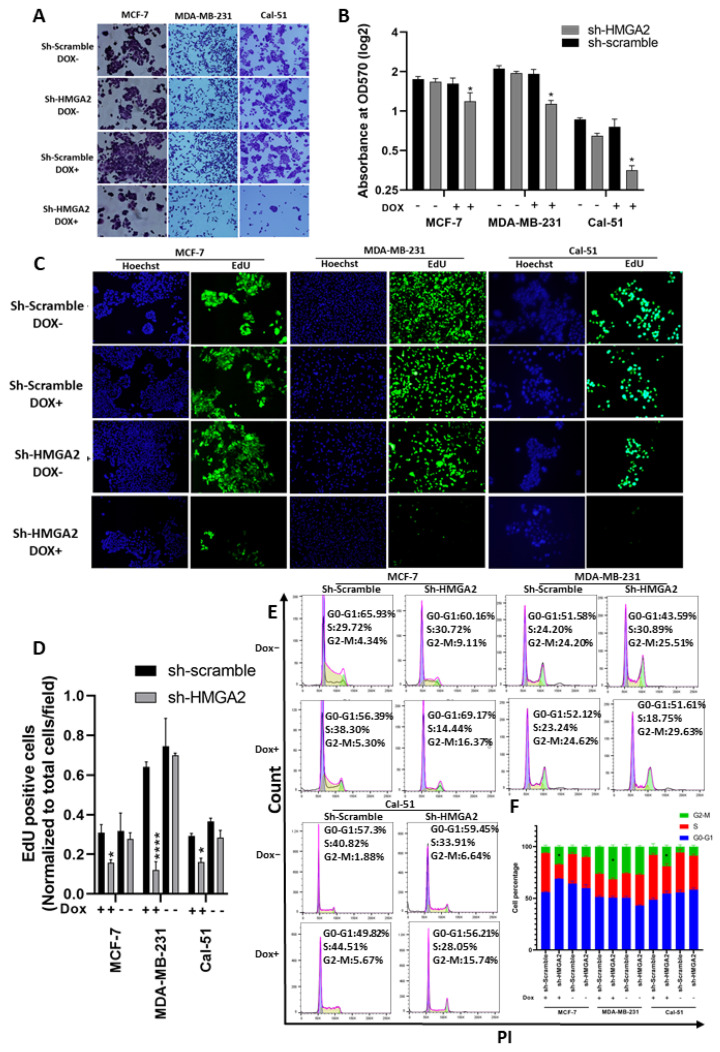
*HMGA2* silencing decreased TNBC proliferation and G2-M arrest. Crystal violet proliferation assay showed a significant reduction in the number and optical density (570 nm) in the HMGA2-silenced (shHMGA2) compared to scrambled control (shScrambled) transduced cells (**A**,**B**). In addition, HMGA2 silencing caused a significant reduction in EdU-positive TNBC cells as determined by fluorescence immunocytochemistry (**C**,**D**). HMGA2 silencing further increased G2-M arrest in both TNBC cell lines compared to shScramble group as shown by propidium iodide DNA content cell cycle analysis (**E**,**F**). The results are shown as mean±SD (*n* = 3), * *p* < 0.05, **** *p* < 0.0001 by *t*-test.

Cell growth analysis performed by crystal violet and EdU assays confirmed our previous results for the MDA-MB-231 cell line, showing reduced cell growth upon knockdown of *HMGA2* (Figure 3A–D) and validated our model system. In addition, the TNBC cell line Cal-51 was found to be highly growth inhibited upon *HMGA2* knockdown. A similar effect was seen for the luminal cell line MCF-7, demonstrating that the role of HMGA2 in supporting cell growth extends beyond TNBC (Figure 3A–D). Cell cycle FACS analysis showed significant arrest in the G2-M phase in MDA-MB-231 (29.63% ± 1.3%), Cal-51 (15.74% ± 1.1%) and MCF-7 (17.285% ± 1.29%) (Figure 3E,F). These studies validated the role of HMGA2 in supporting the proliferation of TNBC cells.

**Figure 4:**
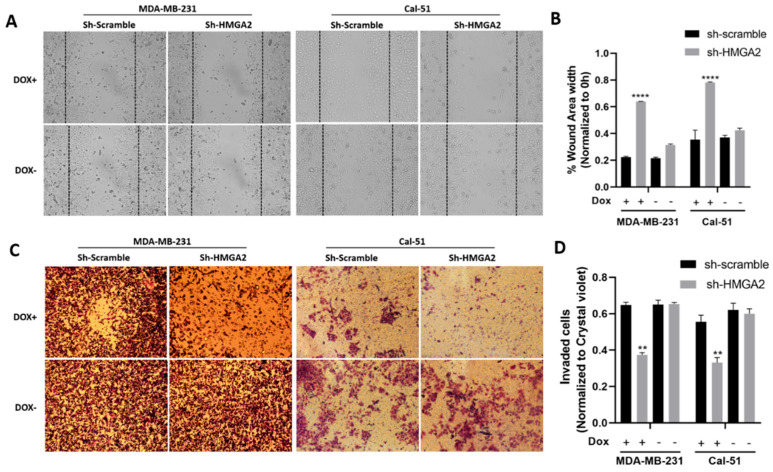
*HMGA2* silencing decreases the invasion and migration of TNBC cells. Micrograph showing reduced invasion of HMGA2-silenced TNBC cells compared to corresponding TNBC control cells (**A**). The number of HMGA2-silenced migrated cells were quantified and compared to shScramble cells for both MDA-MB-231 and Cal-51 cell lines after 48 h treatment with or without doxycycline (Dox) (**B**). Micrograph showing that HMGA2-silenced TNBC cells exhibit reduced invasion (decreased cell migration into the wound gap area) compared to corresponding TNBC control cells (Magnification, ×20) (**C**). The wound area width was quantified and compared to that of shScramble cells for both cell lines (**D**). The results are shown as mean ± SD (*n* = 3) versus shScramble cells, ** *p* < 0.01, **** *p* < 0.0001 by *t*-test.

We next evaluated the role of HMGA2 in invasion, migration and stemness using the newly developed HMGA2-silenced TNBC models. Invasion and migration were evaluated using scratch and transwell matrigel assays. The results of the scratch wound healing assay showed a significant increase in wound area width in cell samples with silenced HMGA2 compared to cell samples transduced with non-targeting shRNA (Figure 4A,B). This was consistent for both the MDA-MB-231 and Cal-51 TNBC cell models. In addition, the invasion assay showed significant inhibition of the invasion of both cell lines upon HMGA2 silencing (Figure 4C,D), demonstrating a role for HMGA2 in supporting TNBC invasiveness.

**Figure 5:**
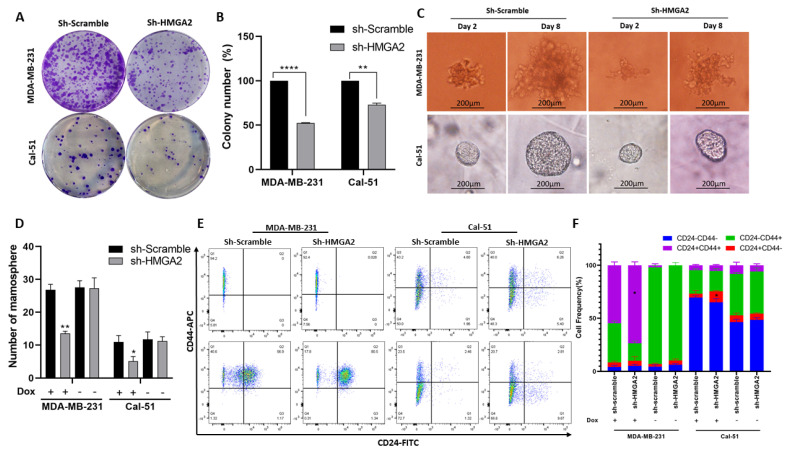
*HMGA2* silencing decreases stemness of TNBC cells. A colony formation assay showed a significant decrease in colony numbers in cells with doxycycline (DOX)-induced HMGA2 knockdown (shHMGA2) (**A**). Colony numbers quantified and compared to shScramble group in both TNBC cell lines (*n* = 3) (**B**). Micrograph showing the size of mammospheres (**C**) and bar graph showing the number of mammospheres (**D**), which were reduced upon HMGA2 knockdown in both TNBC cell lines. The fraction of CD44+CD24+ in MDA-MB-231 and the population of CD44-CD24+ in Cal-51 increased after HMGA2 silencing compared to shScramble cells (**E**,**F**). The results are shown as mean±SD (*n* = 3) versus the shScramble group, * *p* < 0.05, ** *p* < 0.01, **** *p* < 0.0001 by *t*-test.

We also investigated the effect of *HMGA2* silencing on the cancer stem cell characteristics of MDA-MB-231 and Cal-51 TNBC cells. The ability to form colonies was significantly decreased in cells with HMGA2 silencing compared to cells with non-targeting control (Figure 5A,B). In addition, the sizes and numbers of mammospheres were diminished in HMGA2-silenced TNBC cell cultures compared to corresponding scrambled transduced control cell cultures (Figure 5C,D) in both the MDA-MB-231 and Cal-51 cell models. The population of CD44+CD24+ increased significantly in MDA-MB-231 shHMGA2 group (*p*-Value < 0.05), in contrast with the population of CD44-CD24+, which significantly increased in the Cal-51 shHMGA2 group compared to the scramble group (*p*-Value < 0.05) (Figure 4E,F) indicating that HMGA2 supports key cancer stem cell features of TNBC. However, these results should be interpreted with caution since CD44 expression was also affected in controls (i.e., cells with doxycycline treatment/induction of scrambled shRNA).

### 3.3. HMGA2 Silencing Affects NF-kβ/IL-6/STAT3 Signaling

**Figure 6:**
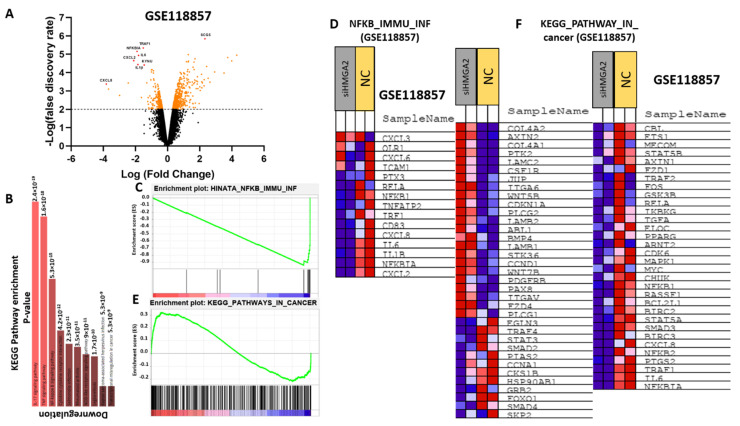
HMGA2 expression correlates with NF-kB signaling in TNBC. Comparative expression profiling of MDA-MB-231 cells treated with HMGA2-specific siRNA or scramble was analyzed using data from GSE 118857. A volcano plot of differentially expressed (DE) mRNAs, adjusted for fold-change>2 and *p* < 0.01, between HMGA2 siRNA treated and negative control (Mock) treated cells (**A**). Gene enrichment showed significant alteration of the NF-kB signaling pathway upon HMGA2 silencing (*p* = 8.9 × 10^−8^) (**B**). Gene set enrichment analysis (GSEA) showed that NF-kB (**C**,**D**) and Cancer pathway (**E**,**F**) gene sets were specifically enriched among the genes differentially expressed between the HMGA2-silenced and scramble groups.

HMGA2 is a transcription factor and may support cancer phenotypes by affecting the transcriptional landscape. To examine this, we analyzed a publicly available RNA-sequencing dataset comparing MDA-MB-231 cells transfected with HMGA2 or scrambled siRNA (GSE118857). Pathway analysis of the 728 differentially-expressed genes demonstrated that *HMGA2* expression was associated with increased activity of the NF-kB (*p* = 8.9 × 10^−8^), TNF (*p* = 2.1 × 10^−7^), IL-17 (*p* = 2.5 × 10^−6^), and NOD-like receptor signaling pathways (*p* = 3 × 10^−6^) (Figure 6A,B). In addition, gene set enrichment analysis (GSEA) showed that *HMGA2* expression was associated with the expression of genes of the NF-kB and general cancer pathways (Figure 6C,D). Many of the genes involved in the NF-kB signaling pathway were highly reduced in HMGA2-silenced cells, including *CXCL2*, *NF-kBIA*, *IL-1b*, *IL-6*, *CXCL8*, *CD83*, *IRF1*, *TNFAIP2*, and *NF-kB1* (Figure 6D). Some of these genes were also present in the general cancer pathway, including *NFkBIA*, *IL-6* and *CXCL8* (Figure 6F). Together, this comparative transcriptome profile suggests that HMGA2 may be a regulator of NF-kB signaling.

**Figure 7:**
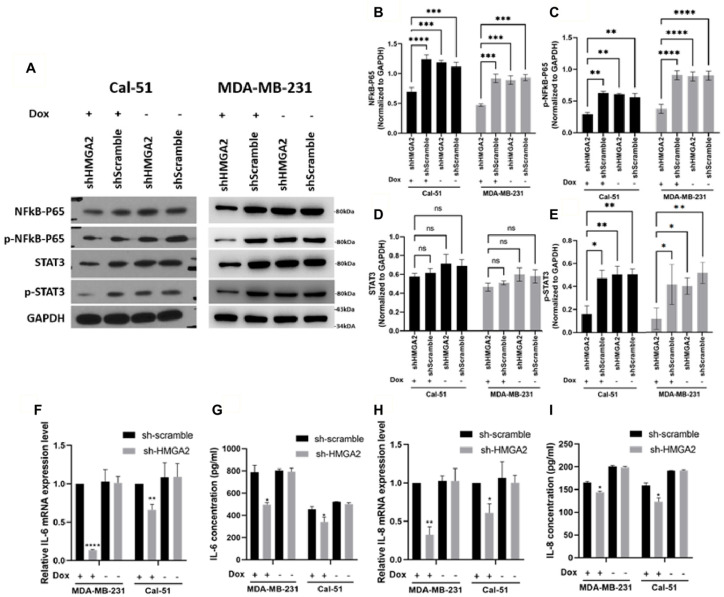
HMGA2 silencing decreased activation of NF-kB/IL-6/STAT3 signaling. Western blotting showed that doxycycline (Dox)-induced HMGA2 silencing (shHMGA2) decreased both NF-kB-p65 (**A**,**B**), *p*-NF-kB-p65 (**A**,**C**) and p-STAT3 (**A**,**E**) but not STAT3 (**A**,**D**) protein levels. In addition, HMGA2 silencing decreased *IL-6* (**F**,**G**) and *IL-8* (**H**,**I**) in both the mRNA and protein levels (*n* = 3). * *p* < 0.05, ** *p* < 0.01, *** *p* < 0.001, and **** *p* < 0.0001 by *t*-test versus the shScramble group.

To confirm that HMGA2 indeed regulates NF-kB signaling in TNBC cells, we investigated and confirmed reduced levels of total NF-kB-P65 (Figure 7A,B) and its phosphorylated form (Figure 7A,C) upon HMGA2 knockdown in our two TNBC cell models. Since NF-κB was shown to regulate the expression of cytokines and growth factors, including IL-6 and IL-8 [19], we evaluated the mRNA and protein levels of IL-6 (Figure 7F,G) and IL-8 (Figure 7H,I) and showed that both were decreased upon HMGA2-silencing. NF-κB-induced IL-6 and IL-8 expression was shown to activate STAT3 [20,21], and in agreement with this, we found that phosphorylation of STAT3 was significantly decreased in the HMGA2-silenced cells compared to scramble control cells (Figure 7A,E), and we did not see any change in total STAT3 protein expression level (Figure 7A,D).

### 3.4. HMGA2 Silencing Decreased IL-6 Levels in TNBC Xenograft Tumors

**Figure 8:**
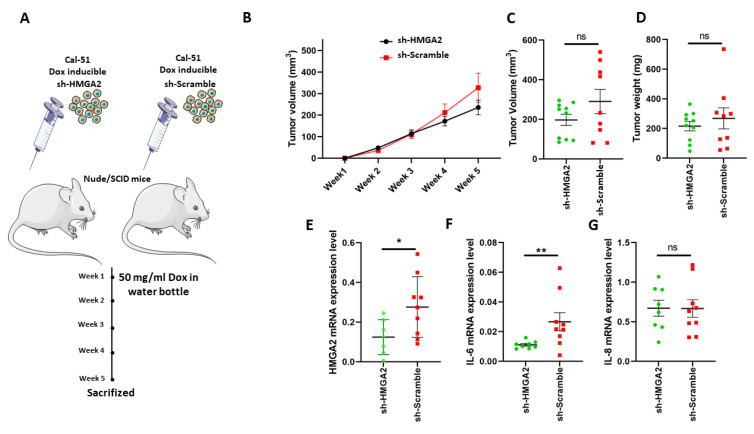
*HMGA2* silencing decreased IL-6 expression in TNBC xenografts. Schematic illustration of an in vivo experiment with *HMGA2*-silenced Cal-51 cells (**A**). Tumor volume was examined weekly (**B**). Tumor volume (**C**) and weight (**D**) after tumor extraction at week 5 were also determined. mRNA expression of *HMGA2* (**E**), *IL-6* (**F**) and *IL-8* (**G**) was measured in tumors, showing significantly reduced *HMGA2* and *IL-6* levels in *HMGA2*-silenced Cal-51 cells. The results are shown as mean ± SD (*n* = 3) versus the shScramble group and statistical analysis was performed using the nonparametric Mann Whitney test. * *p* < 0.05, and ** *p* = 0.01.

Based on the above results, we speculated that *HMGA2* expression might affect TNBC tumor formation. To test this, we implanted Cal-51 cells with doxycycline-inducible expression of HMGA2-specific or non-targeting shRNA in the mammary fat pad of NOG mice (*n* = 8 per group) and provided doxycycline in the drinking water. Tumor sizes were measured weekly for 5 weeks (Figure 8A) and upon resection after the mice were sacrificed. Although there was no statistically significant difference between the groups with and without *HMGA2* knockdown, there was a tendency towards tumors with reduced HMGA2 levels being smaller (Figure 8B–D). Knockdown of *HMGA2* in the tumors with HMGA2 shRNA expression was confirmed with qPCR (Figure 8E). Interestingly, *IL-6* expression was highly reduced in the *HMGA2* knockdown tumors (Figure 8F), confirming our in vitro results. No significant difference in IL-8 mRNA levels between the two groups was observed (Figure 8G).

## 4. Discussion

Compared to other breast cancer subtypes, TNBC is highly aggressive and patients often experience early relapse [22]. Therefore, investigation of molecular mechanisms involved in the etiology of TNBC is critical for gaining increased biological insight into the disease and potentially develop novel treatments. There is increasing evidence linking HMGA2 to tumor initiation, development and metastasis, and this protein was shown to promote cancer development in the breast [6,11,14], colon [9], lung [23] and ovary [24]. HMGA2 binds to the AT-rich sequence of DNA and promotes proliferation and metastasis by regulation of cell cycle, EMT and DNA damage/repair-dependent genes [8]. Thus, HMGA2 may play an important role in the development and progression of TNBC.

Here, we show that TNBC exhibits lower *HMGA2* promoter methylation, and higher HMGA2 mRNA and protein levels compared to normal tissue. This suggested that HMGA2 is overexpressed in TNBC due to promoter demethylation. Treatment of TNBC cell lines with methyltransferase inhibitor did not result in significant changes in HMGA2 expression levels compared to vehicles, but the HMGA2 promoter may already be dethylated in these cell lines of which at least two express HMGA2. Further, high HMGA2 expression was associated with poor prognosis in TNBC, in agreement with our findings that HMGA2 supports several hallmarks of cancer in TNBC cells. HMGA2 expression was demonstrated to be important for the proliferation of TNBC cells. Our cell cycle analysis revealed a significant arrest of G2/M in cells with stable HMGA2 knockdown, supporting our previous results showing that HMGA2 overexpression enhanced proliferation in breast cancer cells [11]. Knockdown of HMGA2 in the luminal cell line MCF-7 also affected cell proliferation, suggesting that the tumor-promoting functions of HMGA2 are not restricted to TNBC. In agreement, other studies have reported that HMGA2 increases cancer cell proliferation in different cancer types [8]. However, in contrast to our results, some studies showed that HMGA2 silencing leads to G0/G1- or S-phase arrest. This suggests that the role of HGMA2 in cell cycle regulation is complex [24,25], which is supported by the finding that HMGA2 affects several key cell cycle regulators such as cyclin-A, -B2, -D, -E and pRB phosphorylation [8].

In previous studies, we demonstrated that HMGA2 increased the invasion and migration of breast and lung cancer cells. We showed that HMGA2 could regulate E-cadherin, snail-1 and vimentin as the most important genes involved in epithelial-to-mesenchymal transition [11,23]. In addition, we showed that targeting HMGA2 with miR-330 and miR-142-3p decreased migration by downregulation of smad3, e-cadherin, snail1, and decreased the phosphorylation of STAT3, AKT and ERK [26,27]. In agreement with these results, the present study demonstrated that HMGA2-silencing significantly reduces the invasion and migration properties of TNBC cells.

We also found that HMGA2-silencing in TNBC cells decreased colony formation ability and reduced the size and number of mammospheres. In addition, we found HMGA2 knockdown increased CD24+ CD44+ breast cancer in moderate HMGA2 expressed cell line and increased CD24+ CD44- in HMGA2 overexpressed cell lines. This is consistent with prior research findings that HMGA2 induces sphere formation and promotes the expression of cancer stem cell markers, including CD44, ALDH1, CD133 in gastric and breast cancer [11,28]. Despite these promising results potentially implicating HMGA2 in tumor formation, we were not able to confirm this using a TNBC xenograft model with HMGA2 knockdown. However, a trend towards lower tumor growth in the HMGA2 knockdown group appeared late in the experiment. Significant differences might have been achieved with better in vivo knockdown or extending the length of the experiment (which was terminated due to reaching maximal allowed tumor size).

**Figure 9:**
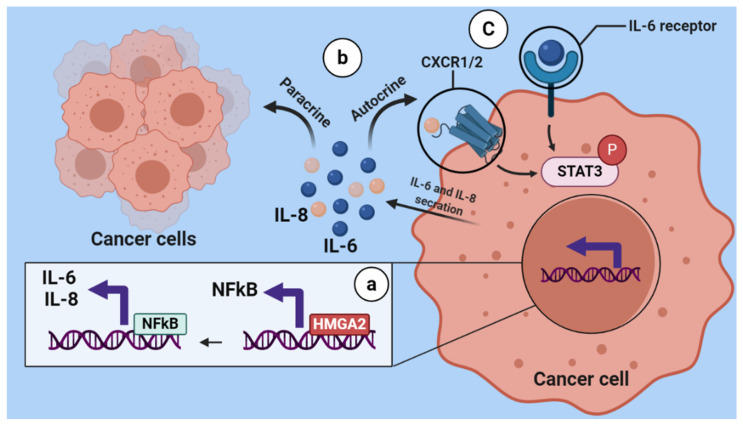
Schematic illustration of regulation of HMGA2 on the NF-κB/IL-6/IL-8/STAT3 axis. HMGA2 as DNA binding protein increases the expression of NFkB, then NFkB increases the secretion of IL-6 and IL-8 from breast cancer cells (a). IL-6 and IL-8 which are released from tumoral cells can bind to their receptor in an autocrine and paracrine manner (b). Subsequently, this binding causes STAT3 signaling activation (C).

NF-κB-signaling pathway overactivation was reported in several tumor tissues such as breast cancer [29,30] and was shown to mediate tumor-cell proliferation, survival and angiogenesis through controlling the expression of genes that support these phenotypes, such as *TNFA, IL6, BCLXL, BCL2, BCLXS, CXCL8,* and *VEGF* [31]. Similarly, we found that HMGA2- silencing in TNBC cells decreased the expression of genes involved in the NF-κB pathway, and the levels of NF-kB p65, phosphorylated NF-kB p65 and phosphorylated STAT3 decreased after HMGA2 silencing. Importantly, it was demonstrated that NF-kB controls the transcription of proinflammatory cytokines, including IL-6 and IL-8 [32]. Accordingly, our results also showed decreased IL-6 and IL-8 after HMGA2 knockdown in TNBC cells. IL-6 and IL-8 are inflammatory cytokines that are produced and secreted by various cells types, including immune cells and tumor cells. They are involved in the proliferation, differentiation and metastasis of malignant cells and are found in high concentrations in serum and tumor tissues of a majority of cancers, including breast cancer [33,34]. It was reported that high expression of both IL-6 and IL-8 are critical for the growth of TNBCs [35], and upregulation of proinflammatory cytokines, including IL-6 and IL-8, in serum is associated with a low survival rate and poor prognosis in breast cancer patients [36]. IL-6 released from malignant cells increases cancer cell growth in an autocrine and paracrine manner, which is important for survival and progression of cancer [37]. In addition, the evidence reveal IL-6 and IL-8 plays a critical role in synergistic signaling involve in a metastatic cascade [35,38,39]. In addition, these cytokines improve malignant cell proliferation and migration through cell-autonomous, autocrine or paracrine mechanisms driven, in part, by the increase in local cell density [38]. Thus, our study indicates that HMGA2 might promote TNBC progression by activation of the NF-kB pathway and subsequent increase in the expression and release of IL-6 and IL-8. These two interleukins can further activate STAT3 signaling in an autocrine and paracrine manner (Figure 9).

## 5. Conclusions

Our results indicate that HMGA2 supports oncogenic features of TNBC cells and may be an important therapeutic target of TNBC since its silencing attenuates cell proliferation, invasion, migration and stemness. These HMGA2-associated phenotypes may be mediated by activation of the NF-kB/IL-6/ IL-8/STAT3 axis. Although further studies are essential to verify the critical mechanisms underlying HMGA2 oncogenesis in TNBC, this study potentially opens new paths of scientific inquiry for HMGA2-targeted therapy in TNBC.

## Data Availability

The data presented in this study are available on request from the corresponding author.

## Supplementary Materials

The following are available online at https://www.mdpi.com/article/10.3390/cancers13205197/s1, Table S1: Immunohistochemistry staining for HMGA2 in normal and TNBC tissues.
