# Correction: Mansoori et al. HMGA2 Supports Cancer Hallmarks in Triple-Negative Breast Cancer. *Cancers* 2021, *13*, 5197

**DOI:** 10.3390/cancers16203444

**Published:** 2024-10-11

**Authors:** Behzad Mansoori, Mikkel Green Terp, Ali Mohammadi, Christina Bøg Pedersen, Henrik Jørn Ditzel, Behzad Baradaran, Morten Frier Gjerstorff

**Affiliations:** 1 Immunology Research Center, Tabriz University of Medical Sciences, Golghasht St., Tabriz 51666-14731, Iran; b.mansoori_lab@yahoo.com (B.M.); baradaranb@tbzmed.ac.ir (B.B.); 2 Department of Cancer and Inflammation Research, Institute for Molecular Medicine, University of Southern Denmark, J. B. Winsløws Vej 25, 3, DK-5000 Odense C, Denmark; mterp@health.sdu.dk (M.G.T.); amohammadi@health.sdu.dk (A.M.); cbpedersen@health.sdu.dk (C.B.P.); hditzel@health.sdu.dk (H.J.D.); 3 Aging Research Institute, Physical Medicine and Rehabilitation Research Center, Tabriz University of Medical Sciences, Golghasht St., Tabriz 51656-65811, Iran; 4 Department of Oncology, Odense University Hospital, DK-5000 Odense C, Denmark; 5 Academy of Geriatric Cancer Research (AgeCare), Odense University Hospital, DK-5000 Odense C, Denmark

## Error in Figures

In the original publication [1], there was a mistake in Figures 4A,C and 7A as published. Duplicated images due to errors in our figure preparation process. The corrected Figure 4A,C and Figure 7A appear below. Additionally, the associated supplementary figure has been updated to reflect this correction. The authors apologize for any inconvenience caused and state that the scientific conclusions are unaffected. This correction was approved by the Academic Editor. The original publication has also been updated.

## Figures and Tables

**Figure 4:**
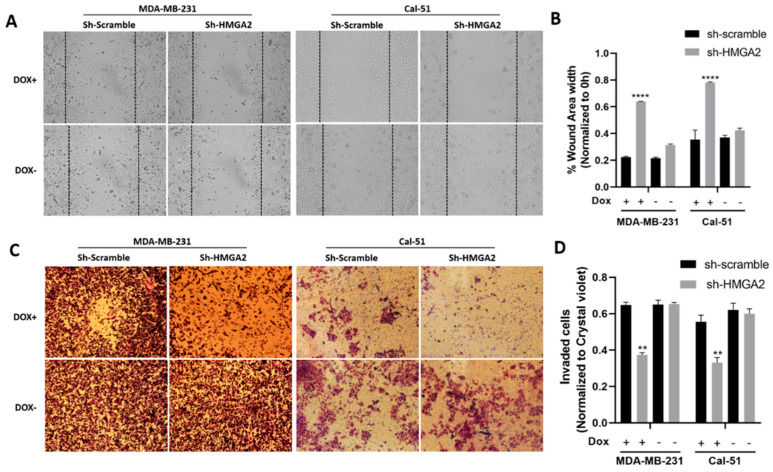
*HMGA2* silencing decreases the invasion and migration of TNBC cells. Micrograph showing reduced invasion of HMGA2-silenced TNBC cells compared to corresponding TNBC control cells (**A**). The number of HMGA2-silenced migrated cells were quantified and compared to shScramble cells for both MDA-MB-231 and Cal-51 cell lines after 48 h treatment with or without doxycycline (Dox) (**B**). Micrograph showing that HMGA2-silenced TNBC cells exhibit reduced invasion (decreased cell migration into the wound gap area) compared to corresponding TNBC control cells (Magnification, ×20) (**C**). The wound area width was quantified and compared to that of shScramble cells for both cell lines (**D**). The results are shown as mean ± SD (*n* = 3) versus shScramble cells, ** *p* < 0.01, **** *p* < 0.0001 by *t*-test.

**Figure 7:**
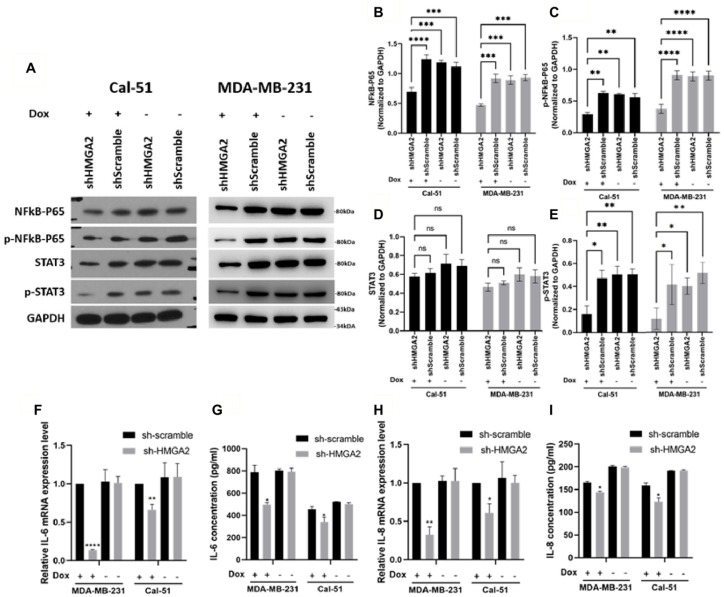
HMGA2 silencing decreased activation of NF-kB/IL-6/STAT3 signaling. Western blotting showed that doxycycline (Dox)-induced HMGA2 silencing (shHMGA2) decreased both NF-kB-p65 (**A**,**B**), *p*-NF-kB-p65 (**A**,**C**) and p-STAT3 (**A**,**E**) but not STAT3 (**A**,**D**) protein levels. In addition, HMGA2 silencing decreased *IL-6* (**F**,**G**) and *IL-8* (**H**,**I**) in both the mRNA and protein levels (*n* = 3). * *p* < 0.05, ** *p* < 0.01, *** *p* < 0.001, and **** *p* < 0.0001 by *t*-test versus the shScramble group.
