# Supplementary material for: HMGA2 Supports Cancer Hallmarks in Triple-Negative Breast Cancer
Source: Cancers (Basel). 2021 Oct 16;13(20):5197. doi: 10.3390/cancers13205197 (PMC8533747; doi:10.3390/cancers13205197)
Supplement: Supplementary file 1 [file cancers-13-05197-s001.zip › supplementary blots.pdf]

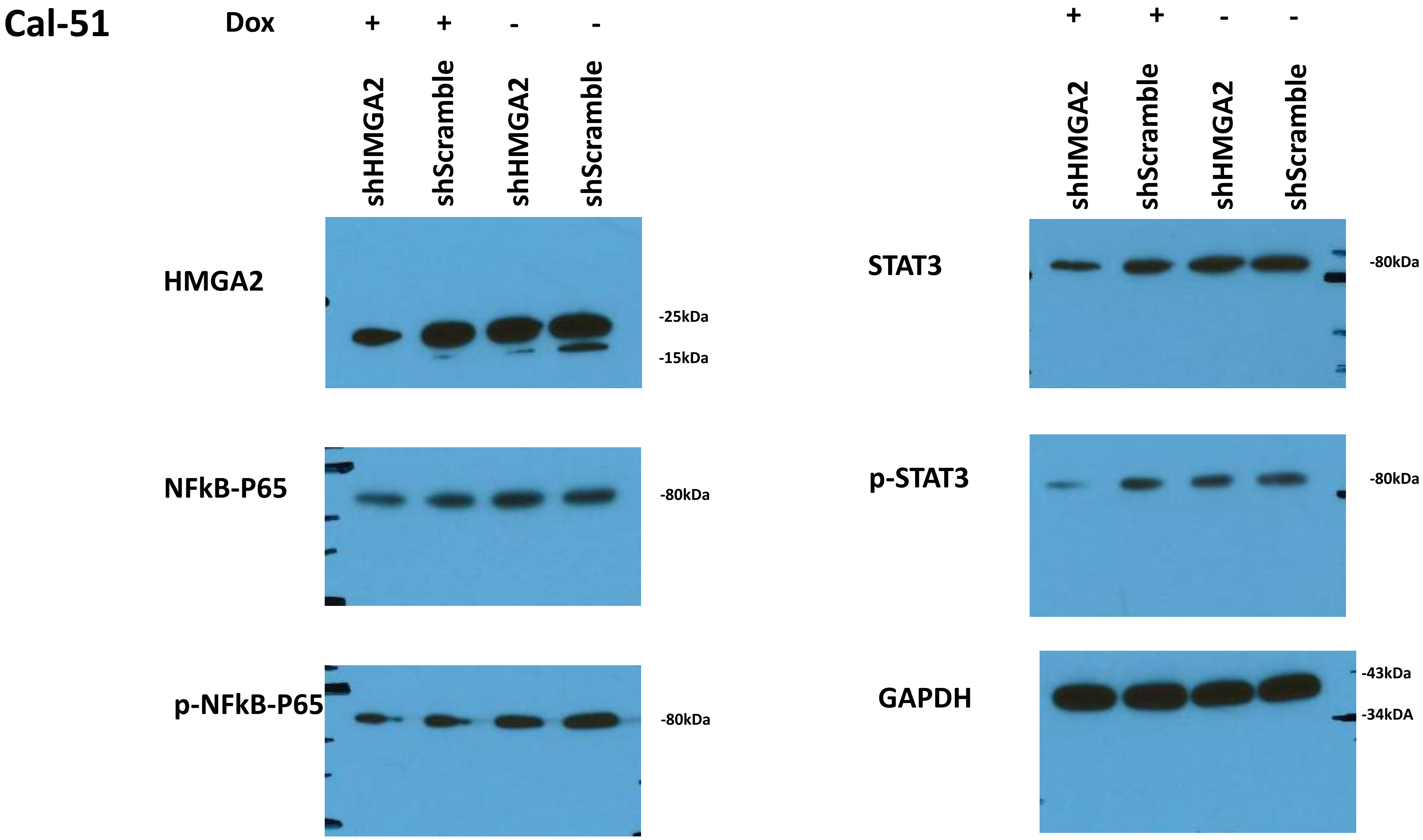
Cal-51
Dox
+
+
-
-
shHMGA2
shScramble
shHMGA2
shScramble
HMGA2
-25kDa
-15kDa
NFkB-P65
-80kDa
p-NFkB-P65
-80kDa
STAT3
-80kDa
p-STAT3
-80kDa
GAPDH
-43kDa
-34kDA

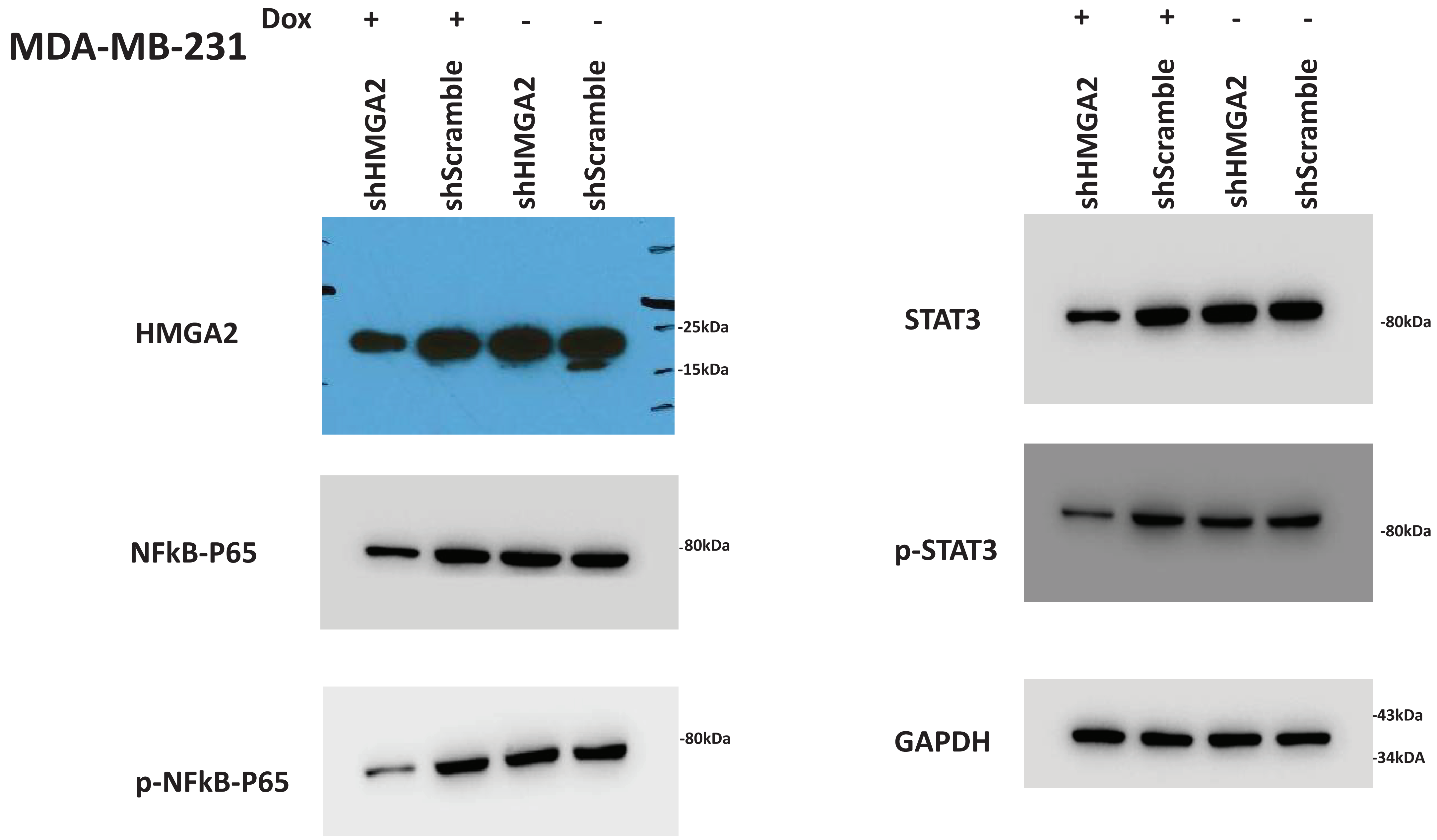

MDA-MB-231
Dox
+
+
-
-
shHMGA2
shScramble
shHMGA2
shScramble
HMGA2
-25kDa
-15kDa
NFkB-P65
-80kDa
p-NFkB-P65
-80kDa
STAT3
-80kDa
p-STAT3
-80kDa
GAPDH
-43kDa
-34kDA
